# Head and neck cancer

**DOI:** 10.1186/1753-6561-7-S2-K12

**Published:** 2013-04-04

**Authors:** Luiz Paulo Kowalski

**Affiliations:** 1 Head and Neck Surgery and Otorhinolaryngology Department, Hospital A C Camargo, National Institute of Science and Technology in Oncogenomics, São Paulo, Brazil

## 

More than 600,000 head and neck cancers (HNC) are diagnosed every year and the incidence and mortality rates vary according to the economical status of the country. Most are oral, pharyngeal or laryngeal squamous cell carcinomas. The most significant risk factors include lifestyle habits (tobacco, alcohol drinking and diet). More recently, exposure to some biological agents (HPV and EBV) and genetic susceptibility were also identified. Tobacco smoking increases acetaldehyde burden following alcohol consumption, and alcohol consumption enhances the activation of pro-carcinogens by induction of cytochrome P450-2E1 system. HPV and EBV are associated with the oropharyngeal and nasopharyngeal cancers, respectively. Genetic susceptibility has a significant role in non-smoking young patients and it is based on differences in the efficiencies of metabolizing carcinogens, DNA repair and cell cycle control. Familial aggregation of HNSCC has been recently reported and the responsible genes are under investigation. Possibly, the identification of a genetic risk profile may improve prevention, early diagnosis and treatment. Most HNC are characterized by local tumor invasion, frequent regional lymph nodes metastasis, and multiple primary cancers in the upper aerodigestive tract, esophagus and lung. Most patients had advanced-stage disease at diagnosis and current treatment options frequently incur significant morbidity. The significant prognostic factors in HNC patients are TNM stage, tumor site and histologic variables. However, tumors of the same site with similar histology and stage may behave differently due to their differing biological characteristics. There is a major interest in the identification of biomarkers (‘‘biological fingerprints”) that could be used in the clinical practice in order to better prognosticate and risk-stratify patients and predict treatment response. The results of these investigations can completely change clinical practice and result in effective “personalized medicine”.

Only HPV positivity and EGFR overexpression are currently considered reliable predictive markers for therapeutic response in HNC patients. Some recently published studies have also shown that TP53 genetic polymorphisms and mutations influence tumor progression and response to therapy. Genotyping has being also utilized to probe other known gene mutations and can offer potential for identification and targeting of key pathways implicated in tumor progression to guide therapeutic strategies. Recently we have investigated the expression profile of Epithelial-Mesenchymal-Transition genes in patients with oral SCC using a cDNA microarray platform coupled to qRT-PCR and immunohistochemical analysis. We identified TWIST1 transcription factor to be overexpressed and to predict metastases and prognosis. Most prognostic information on HNC are patient and tumor-related factors and occasionally pathologic findings. These variables influence outcomes prediction at diagnosis, but do not consider several others such as response to initial therapy, complications, risk of recurrences and second primary tumors. Furthermore, the risks of recurrence, second primary cancers and non-cancer related deaths change during follow-up when a patient develops a recurrence or second primary tumor. More sophisticated prognostic evaluations are ongoing.

## Competing interests

There are no competing interests in this presentation.

